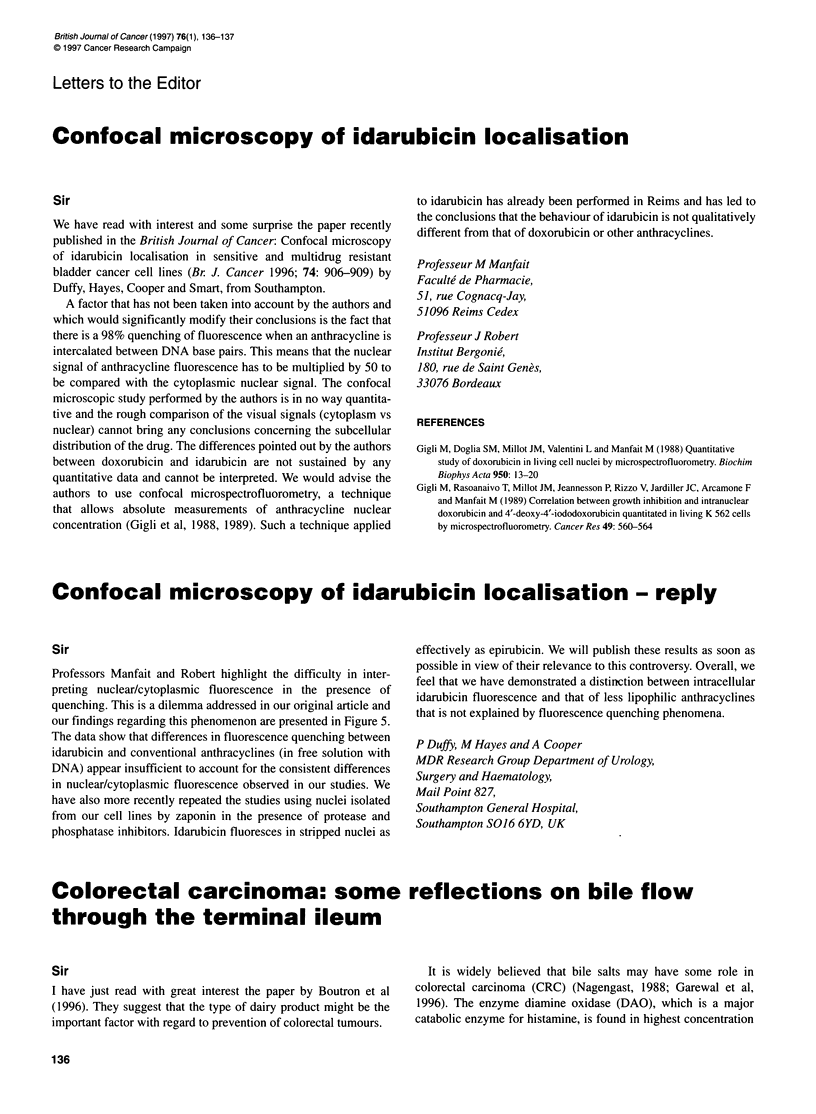# Confocal microscopy of idarubicin localisation.

**DOI:** 10.1038/bjc.1997.349

**Published:** 1997

**Authors:** M. Manfait, J. Robert


British Journal of Cancer (1997) 76(1), 136–137
© 1997 Cancer Research Campaign

Letters to the Editor

### Confocal microscopy of idarubicin localisation

### Sir

We have read with interest and some surprise the paper recently published in the *British Journal of Cancer*: Confocal microscopy of idarubicin localisation in sensitive and multidrug resistant bladder cancer cell lines (*Br. J. Cancer* 1996; **74**: 906–909) by Duffy, Hayes, Cooper and Smart, from Southampton.

A factor that has not been taken into account by the authors and which would significantly modify their conclusions is the fact that there is a 98% quenching of fluorescence when an anthracycline is intercalated between DNA base pairs. This means that the nuclear signal of anthracycline fluorescence has to be multiplied by 50 to be compared with the cytoplasmic nuclear signal. The confocal microscopic study performed by the authors is in no way quantitative and the rough comparison of the visual signals (cytoplasm vs nuclear) cannot bring any conclusions concerning the subcellular distribution of the drug. The differences pointed out by the authors between doxorubicin and idarubicin are not sustained by any quantitative data and cannot be interpreted. We would advise the authors to use confocal microspectrofluorometry, a technique that allows absolute measurements of anthracycline nuclear concentration (Gigli et al, 1988, 1989). Such a technique applied to idarubicin has already been performed in Reims and has led to the conclusions that the behaviour of idarubicin is not qualitatively different from that of doxorubicin or other anthracyclines.

*Professeur M Manfait*
*Faculté de Pharmacie,*
*51, rue Cognacq-Jay,*
*51096 Reims Cedex*

*Professeur J Robert*
*Institut Bergonié,*
*180, rue de Saint Genès,*
*33076 Bordeaux*

### REFERENCES

Gigli M, Doglia SM, Millot JM, Valentini L and Manfait M (1988) Quantitative study of doxorubicin in living cell nuclei by microspectrofluorometry. *Biochim Biophys Acta* **950**: 13–20

Gigli M, Rasoanaivo T, Millot JM, Jeannesson P, Rizzo V, Jardiller JC, Arcamone F and Manfait M (1989) Correlation between growth inhibition and intranuclear doxorubicin and 4′-deoxy-4′-iododoxorubicin quantitated in living K 562 cells by microspectrofluorometry. *Cancer Res* **49**: 560–564